# Room temperature shipment does not affect the biological activity of pluripotent stem cell-derived retinal organoids

**DOI:** 10.1371/journal.pone.0233860

**Published:** 2020-06-01

**Authors:** Maria Georgiou, Valeria Chichagova, Gerrit Hilgen, Birthe Dorgau, Evelyne Sernagor, Lyle Armstrong, Majlinda Lako

**Affiliations:** 1 Biosciences Institute, Faculty of Medical Sciences, Newcastle University, Newcastle upon Tyne, England, United Kingdom; 2 Newcells Biotech Ltd, Newcastle upon Tyne, England, United Kingdom; University of Florida, UNITED STATES

## Abstract

The generation of laminated and light responsive retinal organoids from induced pluripotent stem cells (iPSCs) provides a powerful tool for the study of retinal diseases and drug discovery and a robust platform for cell-based therapies. The aim of this study is to investigate whether retinal organoids can retain their morphological and functional characteristics upon storage at room temperature (RT) conditions and shipment by air using a commercially available container that maintains the environment at ambient temperature. Morphological analysis and measurements of neuroepithelial thickness revealed no differences between control, RT incubated and shipped organoids. Similarly immunohistochemical analysis showed no differences in cell type composition and position within the laminated retinal structure. All groups showed a similar response to light, suggesting that the biological function of retinal organoids was not affected by RT storage or shipment. These findings provide an advance in transport of ready-made retinal organoids, increasing their availability to many research and pharma labs worldwide and facilitating cross-collaborative research.

## 1. Introduction

A new technology, namely the large scale generation of three-dimensional (3D) retinal organoids has emerged by differentiating human embryonic stem cells (hESCs) and human induced pluripotent stem cells (hiPSCs) into “synthetic retinae” [1–3]. These 3D retinal structures contain all major retinal cell subtypes with distinct layering reflecting to a large extent structural, morphological and functional properties of human retina [4]. This approach has been used to provide patient specific disease models for better understanding of human retinal disease, to generate novel insight into human retinal development, to reveal unknown mechanisms of pathogenesis and to provide new avenues for drug screening and cell-based replacement therapies [5].

While organoid technology is available to some specialised labs, their generation would involve considerable expertise and infrastructure for some others, hence transportation of well characterised retinal organoids, will ultimately make this technology more accessible globally. Shipping conditions are very critical for the tissue and they depend on temperature control and timing [6]. Transportation of retinal organoids should ensure that the organoids are shipped immediately using the shortest route possible, in order to retain survival, cell type composition, position and functionality. Therefore, special containers that maintain the temperature, as well as reliable delivery companies should be considered. This is very critical as exposure of tissues to high or low temperatures, or temperature fluctuations could be detrimental, affecting the biological and mechanical activity of the tissue, causing cell injury and intracellular degeneration of cells [6]. Therefore, by monitoring temperature condition the cell damaging effects are minimised. For temperature-controlled shipments, many options are available but not always economically practical [7]. The transportation of tissues depends on the nature of the samples, whether the samples are preserved or live, but also depends on the shipment method, whether this is by air or road transportation [6]. Several published studies have reported the successful transportation of tissues at cooled or frozen conditions using commercially available containers [8, 9]. Specifically, in 2013 a study using cornea and blood revealed the maintenance of cooling temperatures during organ shipment [9]. Another study demonstrated the successful shipment of femoral head tissue and Achilles tendon at -40 °C for at least 48 hours indicating that the safety and quality of human tissue could be maintained during transportation at during shipment [8]. Hori and colleagues have demonstrated a new technology for shipping RPE tissues at room temperature by using a heat insulating container to minimise the physical and chemical stress on the cells [10]. Additionally, a recent study has developed a new protocol for shipping retinal organoids at 37°C to facilitate the preclinical studies in animal models and potentially transplantation by maintaining the viability of the tissue and preventing tissue damage [11].

In this study we determine how storage of retinal organoids and their shipment at room temperature (RT) at normal (0.04%) CO2 levels affects organoid morphology, cellular dynamics and functionality compared to their maintenance in a humidified environment at 37 °C with 5% CO_2_.

## 2. Materials and methods

### 2.1. Pluripotent stem cell culture

Two control hiPSC lines, WT3 (Ad4) [1] and WT4 (MJN1), were cultured in mTeSR^™^1 (StemCell Technologies, 05850) supplemented with penicillin/streptomycin (P/S) (Gibco, 15140–122) on 6-well plates (Corning, NY) pre-coated with reduced growth-factor Matrigel (BD, 354230). Cell culture medium was changed every day and hiPSCs were passaged every 4–5 days using 0.02% Versene EDTA (Lonza BE17-711E) at a ratio of 1:6. Cells were maintained in a humidified environment at 37 °C with 5% CO_2_.

### 2.2. Human iPSC differentiation to retinal organoids

Human iPSCs were differentiated to retinal organoids based on a previously described protocol with minor modifications [12]. Briefly, hiPSCs were initially washed with Phosphate buffered saline (PBS), and dissociated into single cells using Accutase (Gibco, A1110501). The hiPSCs were seeded at a density of 7,000 cells/well onto U-bottom 96-well plates (Helena, 92697T) manually pre-coated with Lipidure solution (AMSbio, AMS.52000011GB1G) in mTeSR™1 with 10 μM Y-27632 ROCK inhibitor (Chemdea). After 2 days, 200 μl of differentiation medium consisting of 41% Iscove’s modified Dulbecco’s medium (IMDM, Gibco, 12440–053), 41% Hams F12 (Gibco, 31765–029), 15% KnockOut serum replacement (KOSR) (Gibco, 10828–028), 1% Glutamax (Gibco, 35050–038), 1% chemically defined lipid concentrate (Thermo, 11905031), 225 μM monothioglycerol (Sigma, M6145), and 1% P/S (Gibco, 15140–122) was added per well followed by half medium changes two days later. Half of the differentiation medium was changed every 2 days. On day 6 of differentiation, 2.25 nM BMP4 (R&D, 314-BP) was added to the media and the medium was replaced every 3 days thereafter. On day 18 the medium was changed to reversal medium using DMEM/F12, 1% Glutamax, 1% N2 supplement (Thermo, A1370701), 4 μM CHIR99021 (Sigma-Aldrich, SML1046-5MG), 2.5 μM SU5402 (Tocris, 3300), and 1% P/S, where the medium was changed every 2 days. At day 24, the medium was replaced to maintenance medium consisting of DMEM/F12 (Gibco, 31330–038), 5% Foetal Bovine Serum (FBS), 1% N2 supplement, 0.1 mM taurine (Sigma, T8691), 0.25 μM retinoic acid (Sigma, R2625), 0.25 μg / ml Fungizone (Gibco, 15290–02), 1% P/S (Gibco, 15140–122), medium was changed every 3–4 days thereafter.

### 2.3. Sectioning of organoids

Prior to sectioning, retinal organoids were collected and fixed in 4% paraformaldehyde (PFA) for 15 minutes (Santa Cruz, 30525-89-4) at RT, followed by three washes with PBS for 5 minutes each. Retinal organoids were dehydrated in 30% sucrose at 4 °C overnight. The retinal organoids were then embedded in moulds (Tebu-Bio, UK 18985–1) in optimum cutting temperature (OCT) medium (Cell Path Ltd., Newtown, UK). The samples were sectioned into slices of 10 μm thickness using a cryostat (Leica, CM1860), and placed by order on glass slides (SuperFrost, Menzel) and stored at -20 °C.

### 2.4. Immunohistochemistry (IHC)

Retinal organoid sections were separated using ImmEdge pen (VectorLabs, H-4000), and air-dried for 20 minutes at RT, followed by three washes of PBS for 5 minutes. Sections were incubated with blocking solution (10% goat/donkey serum, 0.3% Triton-X in PBS) for 1 hour at RT. Primary antibodies (S1 Table) were diluted in antibody diluent (1% bovine serum albumin (BSA, Sigma, A9418), 0.3% Triton-X (in PBS) followed by incubation of samples overnight at 4 °C. Sections were washed thrice with PBS for 15 minutes, and secondary antibodies conjugated to fluorophores (S2 Table) were diluted in antibody diluent, and added to the sections for 1 hour at RT followed by three washes with PBS. Afterwards, sections of retinal organoids were counterstained with Hoechst nuclear stain (Sigma, B2261) diluted in Vectashield at 1:1000 (Vector Laboratories, Burlingame, CA, H-1000). Non-specific staining was controlled by including control groups with secondary antibody only.

#### 2.4.1 TUNEL staining

Slides containing Ad4 and MJN1 retinal organoids sections were incubated initially with PBS and afterwards with proteinase K. The slides were then treated for 10 minutes with 3% H_2_O_2_ at RT. Thereafter, the slides were incubated with reaction buffer for 30 minutes at room temperature. Tunnel assay was then used according to the manufacturer’s instructions (Biovision, Mountain View, CA, USA). Apoptotic and necrotic cells were detected as dark particles using the Axio Imager microscope with Apotome structured illumination fluorescence (Zeiss AxioVert 2 Germany).

### 2.5. RT and shipment of retinal organoids

For the RT experiment, day 360 retinal organoids were stored at RT conditions for five days. The start of the experiment was defined as day -5. Following 5 days of incubation at RT, retinal organoids were transferred to the incubator, at 37 °C with 5% CO_2_; this is defined as day 0. The organoids were kept in the incubator for 15 days before their collection for IHC: this is defined as day 15 of the RT experiment.

The shipment experiment was performed at two different time points during retinal differentiation: day 135 and 160. Prior to shipment, 96-well culture plates of retinal organoid were fed with 100 μl of fresh retinal organoid media prior to shipping. The 96-well plates were covered with parafilm to prevent any leakage and were further sealed with a plastic sealed bag containing an absorbent material, and placed horizontally in a specific shipping container at RT in a controlled ambient environment system (QuickSTAT). The plates were surrounded with cloth/tissue to prevent the movements of the plates and their leakage in the shipping container. A temperature control was activated and placed inside the box according to the manufacturer’s instructions to record the temperature throughout the shipping (S1A Fig).

The parcel was delivered by car to London and then by plane to Frankfurt, Germany at RT at normal CO_2_ levels (0.04%) (S1B Fig). This is defined as day -3 of the shipment experiment. After three days the parcel returned back to the Biosciences Institute at Newcastle via the same route (S1C Fig). The arrival of day 135 and 160 retinal organoids back at the Institute was recorded as day 0. The organoids were placed into the incubator at 37°C upon delivery and maintenance media was replaced every 2 days. Collection of the organoids for IHC experiment performed after their incubation for 7 days to recover.

Control plates with day 135, 160 and day 360 retinal organoids were maintained in a humidified environment at 37 °C with 5% CO_2_ for comparison. These control organoids were collected at the same time points as the RT and shipped organoids.

### 2.6. Microscopy

Digital images were taken using an Axio Imager microscope with Apotome structured illumination fluorescence (Zeiss AxioVert 2 Germany). While approximately, 8–10 organoids were analysed per condition, only five representative images were taken of each condition and used for further IHC analyses. Images are presented as a maximum projection and adjusted for brightness and contrast in Adobe Photoshop (Adobe Systems). Brightfield images of retinal organoids were captured using Axiovision 4.3 (Zeiss), and analysis of the neuroepithelial thickness was performed using the ImageJ software as described previously [13]. Briefly, the thickness of the neuroepithelial was measured by taking 6 measurements across the neuroepithelial of each organoid, using 8–10 representative organoids from each condition. The average number of the neuroepithelial thickness from control and RT organoids was statistically analysed using Prism (GraphPad, USA).

#### 2.6.1. Image quantification

Quantification of retinal cell types was performed using the MATLAB software (Mathworks, MA) as described previously [14]. Using MATLAB’s region props property, information about the size, the length and the average intensity of each retinal cell was calculated, and the total size and percentage of positive cells in relation to nuclear stained cells were exported in an Excel file for additional analysis. Five to six representative images, of whole retinal structure per condition were quantified. The percentage of ciliated cells was calculated as cilia numbers/total cell numbers (DAPI) in putative outer nuclear layer x 100.

### 2.7. Electrophysiological recordings

Electrophysiological recording of shipped retinal organoids was performed as described in Dorgau and Felemban et al., 2019 [14]. Briefly, 24 hours prior to recordings, incubation with 9-cis retinal (10 nM; Sigma-Aldrich, UK) was carried out. Organoids were placed facing down with the presumed retinal ganglion cell (RGC) layer onto a 4096 channel multielectrode array (MEA). BioCam4096 MEA platform with BioChips 4096S+ (3Brain GmbH, Lanquart, Switzerland) was used to extracellularly record the activity of retinal ganglion cells in retinal organoids. Full field white light pulses (WLP, 200 ms, 217 μW/cm2 irradiance, 1Hz) were flashed for 5 minutes onto the organoids following recording spontaneous activity in the dark for 5 minutes. Firing rate analyses were performed by using MatLab (Mathworks, MA) and statistical significance tests (Mann-Whitney test) were evaluated using Prism (GraphPad, CA). Retinal ganglion cells were considered responsive if they changed their spiking activity at least 25% (increase or decrease) during 30 seconds after WLP onset compared to the similar time window before the light stimulus (dark condition).

### 2.8. Transmission electron microscopy (TEM)

Retinal organoids were collected and fixed with 2% gluteraldehyde in 0.1 M sodium cacodylate buffer and kept at 4 °C. The samples were processed at the Newcastle University electron microscopy facility, where 1% of osmium tetroxide was used to fix further the samples followed by dehydration and embedding using gradient acetone, and epoxy resin respectively. Heavy metals (uranyl acetate and lead citrate) were used to stain ultrathin sections (70 nm) on copper grids, imaged on a Philips CM100 TEM with high-resolution digital image capture.

### 2.9. Statistical analyses

Statistical analysis was performed using Prism (GraphPad, USA). Statistical significance was tested using two-tailed Student’s *t* test. Error bars represent standard error of mean (SEM) unless indicated otherwise. Statistical significance of pairwise comparisons is indicated by asterisks: * p-value <0.05, ** p-value <0.01, *** = p-value <0.001. *** = p-value <0.0001.

## 3. Results

### 3.1. Short-term storage of day 360 retinal organoids at RT does not induce phenotypic or structural differences

**Fig 1:**
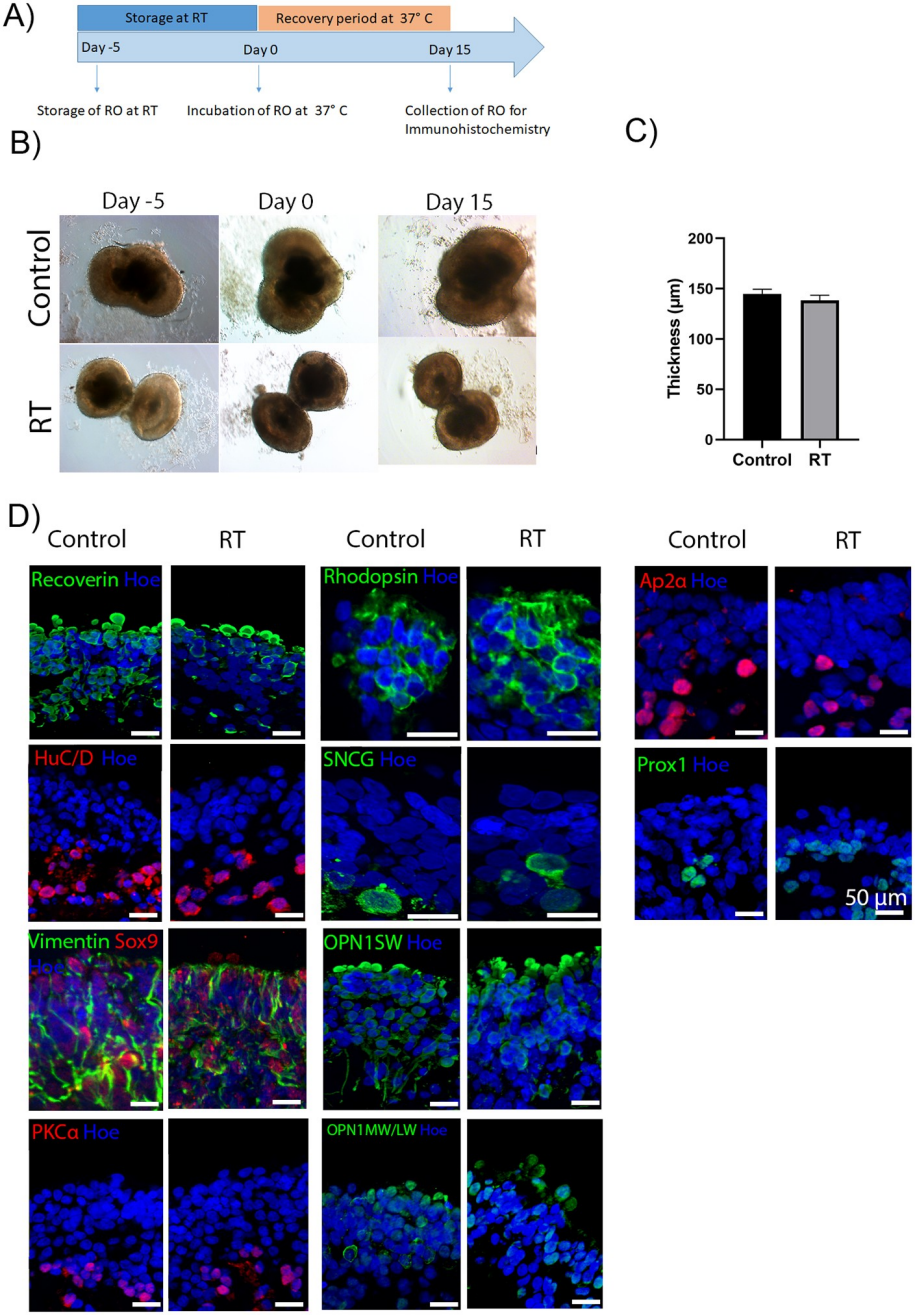
Morphological characteristics of control and RT retinal organoids before and after exposure to RT conditions. (A) Schematic diagram of RT experiment. (B) Representative examples of hiPSCs-derived retinal organoids at day 360 of differentiation. Top images represent control retinal organoids that remained in a humidified environment at 37°C with 5% CO_2_. Bottom images represent retinal organoids that were kept at RT for 5 days. Day -5 represents the first day of storing organoids at RT, day 0 represents the day that the organoids were transferred to the incubator at 37 °C with 5% CO_2_, and day 15 represents the recovery period of the organoids, before their collection, in a humidified environment at 37°C with 5% CO_2_. Scale bars = 100 μm. (C) The thickness of the neuroepithelial layer across the whole organoid was measured in μm using the ImageJ software. Data are shown as mean ± SEM of 8 representative organoids from each group. An unpaired t-test was performed to estimate differences in the neuroepithelial thickness between the control and RT organoids showing no significant differences between the groups (p = 0.3713). (D) Immunohistochemical analysis of retinal markers of control and RT retinal organoids after 15 days of recovery from storage at RT for 5 days. Expression of photoreceptors (Recoverin, green) and amacrine and ganglion cells (HuC/D, red), Müller cells (Vimentin–green, Sox9 -red), bipolar cells (PKCα, red), Rod photoreceptors (Rhodopsin, green), ganglion cells (SNCG, green), amacrine cells (AP2α, red), and S cone photoreceptors (Opsin SW, green), L/M cone photoreceptors (Opsin MW/LW, green) and horizontal cells (Prox1, green). Nuclei are counterstained with Hoechst (Hoe, blue). Scale bar = 50 μm.

Human iPSCs were differentiated into retinal organoids using an established protocol (S2 Fig) [12]. To assess the impact of RT storage, day 360 organoids were kept outside the incubator for 5 days (Fig 1A). Bright field images of the retinal organoids from both day 360 control and RT–kept organoids were taken at day -5, 0, and day 15 of the RT experiment (Fig 1B), showing no morphological changes between the control and RT group. Measurement of bright phase neuroepithelial thickness revealed no significant differences between the two groups (Fig 1C).

**Fig 2:**
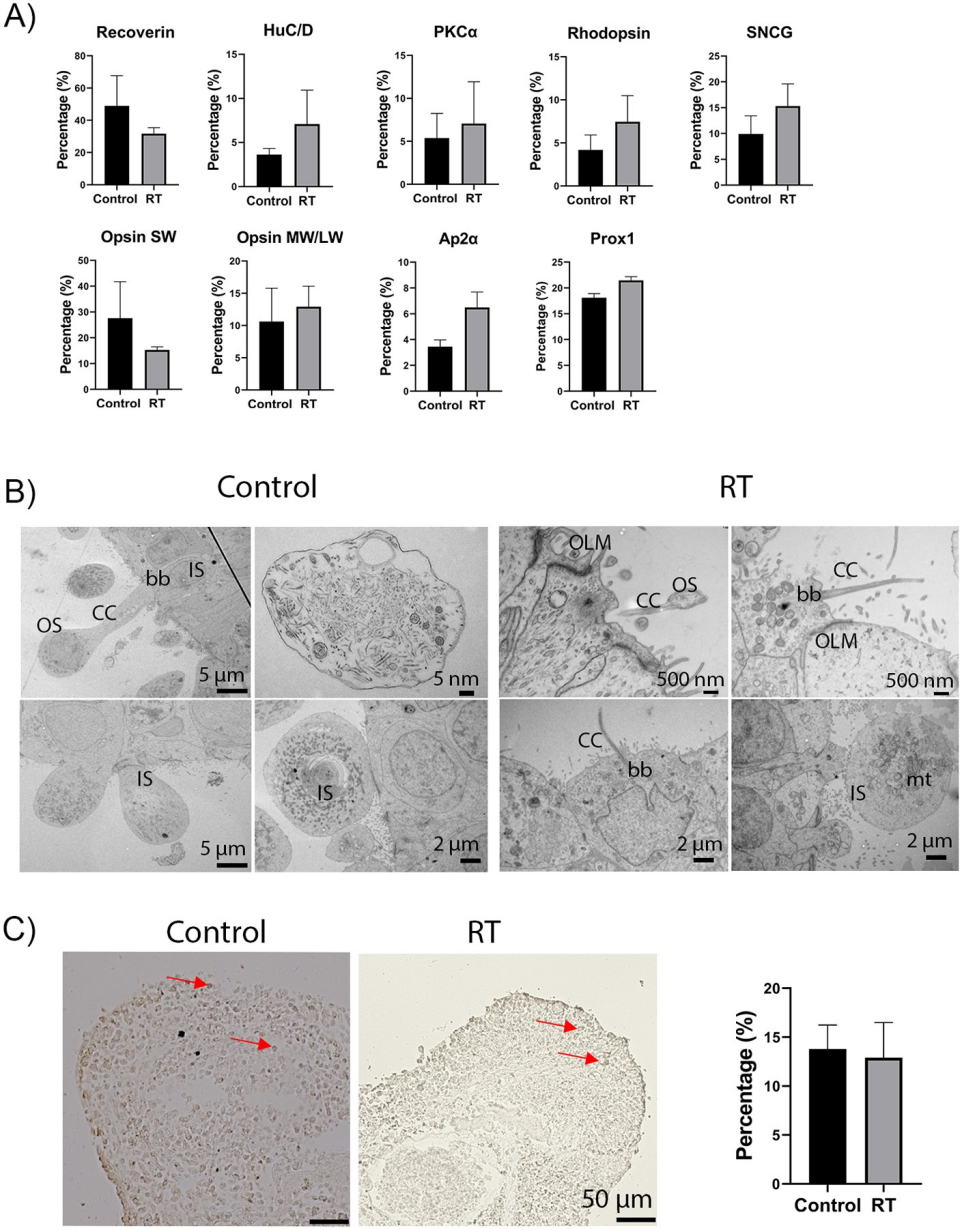
Analysis of day 360 control and RT retinal organoids. (A) Quantification analysis of immunopositive cells in control and RT organoids. Recoverin (p = 0.4766), HuC/D (p = 0.3463), PKCa (p = 0.6996), Rhodopsin (p = 0.1608), SNCG (p = 0.1254), AP2α (p = 0.1506), Opsin SW (p = 0.2012), Opsin MW/LW (P = 0.5941) and Prox1 (P = 0.0624), analysis revealed no significant difference in the percentage of immunoreactive-positive cells between control and RT conditions. Data are shown as mean ± SEM, n = 5 representative images per cell marker were quantified per condition. (B) Ultrastructural analysis of recovered RT and control retinal organoids revealed the presence of photoreceptor outer segments (OS), photoreceptors inner segments (IS) connecting cilium (CC), basal body (bb), outer limiting membrane (OLM), and mitochondria (mt). (C) Brightfield images of apoptotic cells stained with dark brown colour, indicated by red arrows, in control (left image) and RT (right image) condition. Quantification analysis of apoptotic cells was performed showing no significant differences between the two conditions (p = 0.8442). Scale bar = 50 μm.

The morphology and structure of day 360 retinal organoids were evaluated in detail by immunofluorescence staining, which revealed the presence of all key retinal cell types in control and RT conditions (Fig 1D, S3 Fig). The expression of pan photoreceptor marker Recoverin was observed at the apical site of the organoids (Fig 1D, S3 Fig), while HuC/D positive cells, representing ganglion and amacrine cells were mainly located in the basal layer of the organoids (Fig 1D, S3 Fig). Also, immunostaining for gamma synuclein (SNCG), was used to identify the putative retinal ganglion cells (Fig 1D, S3 Fig). All cone types, long wavelength /middle wavelength and short wavelength (LW/MW and SW), were also found in both conditions (Fig 1D, S3 Fig). Rod photoreceptor cells were identified by Rhodopsin at the apical layer of control and RT organoids (Fig 1D, S3 Fig). The presence of Müller glia cells was confirmed by Vimentin staining neurofilaments, which extended throughout the retina, and Sox9 staining the nucleus of Müller glia cells (Fig 1D, S3 Fig). Bipolar and amacrine cells identified by PKCα and AP2α respectively were also identified in both conditions (Fig 1D, S3 Fig). Putative horizontal cells detected with Prox1 antibody were mainly found in the middle layer of the organoids (Fig 1D, S3 Fig). Cell quantification analyses of Recoverin, HuC/D, PKCα, Rhodopsin, SNCG, Opsin MW/LW, AP2α, Prox1, ARL13B, and Opsin SW revealed no significant differences between the two conditions (Fig 2A). TEM of day 360 control and RT organoids revealed the presence of developing outer segments (OS), inner segments (IS), outer limiting-like membrane (OLM), connecting cilia (cc), basal body (bb) and mitochondria (mt) in both conditions (Fig 2B). Additionally, apoptotic and necrotic cells or stress vacuoles were identified in both control and RT condition using Tunel staining (Fig 2C), revealing no significant differences between the two groups. In summary, these results indicate that culturing of retinal organoids for at least 5 days at RT has no effects on their structure and morphology.

### 3.2. Transportation of organoids at RT conditions

**Fig 3:**
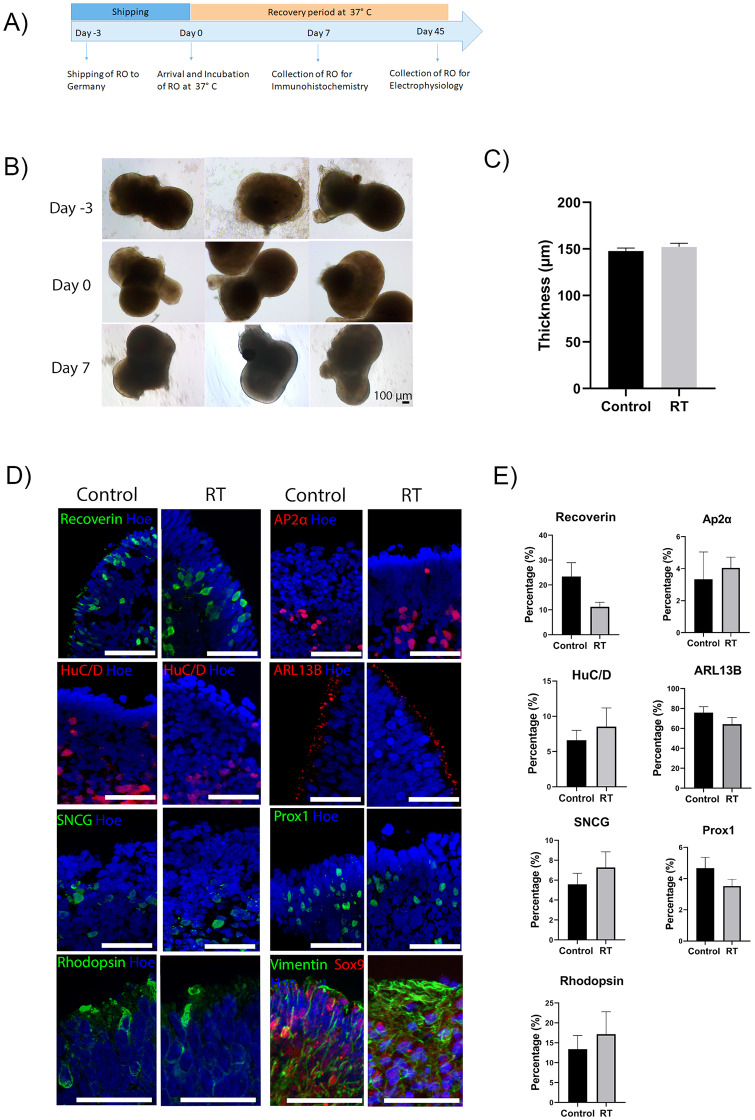
Characterisation of day 135 retinal organoids after shipment. (A) Schematic diagram of shipment experiment. (B) Bright field images of day 135 hiPSCs-derived retinal organoids were captured on day -3, representing the organoids before shipping, day 0, arrival of shipped organoids, and day 7 represents the recovery period of the organoids in a humidified environment at 37°C with 5% CO_2_. Scale bars = 100 μm. (C) The thickness of neuropithelium of day 135 shipped retinal organoids compared to control organoids. The data are shown as mean ± SEM. Statistical analysis was performed using unpaired t-test. No statistically significant differences were observed between the two groups, p = 0.4104. (D) Expression of retinal markers in both control and shipped retinal organoids revealed the presence of photoreceptors (Recoverin, green), Rod photoreceptors (Rhodopsin, green), amacrine cells (AP2α, red), amacrine and ganglion cells (HuC/D, red), ganglion cells (SNCG), connecting cilium (ARL13B red), Müller cells (Vimentin–green, Sox9 –red) and horizontal cells (Prox1, green). Nuclei were counterstained with Hoechst (Hoe, blue). Scale bar = 50 μm. (E) Quantification graphs of Recoverin (p = 0.1120), AP2α (p = 0.7156), HuC/D (p = 0.5570), ARL13B (p = 0.2566), SNCG (p = 0.4259), Prox1 (p = 0.2329) and Rhodopsin (p = 0.5804) revealed no significant difference in the percentage of immunoreactive-positive cells between control and RT conditions. Data are shown as mean ± SEM, n = 3–6 representative images per cell marker were quantified per condition.

Following the successful storage of retinal organoids for 5 days at RT, we investigated whether these could be shipped to another country in a controlled environment (S1A Fig). Thus, organoids aged day 135 and 160 were shipped from our lab in Newcastle to Frankfurt by air and then back to the lab at ambient temperature, using a temperature control to monitor the temperature of the environment (S1A and S1B Fig). Upon arrival in our laboratory, the organoids were placed in incubator for 7 days and compared to the control group, which was maintained in standard culture conditions (Fig 3A).

#### 3.2.1. Comparison of control and shipped organoids at day 135

**Fig 4:**
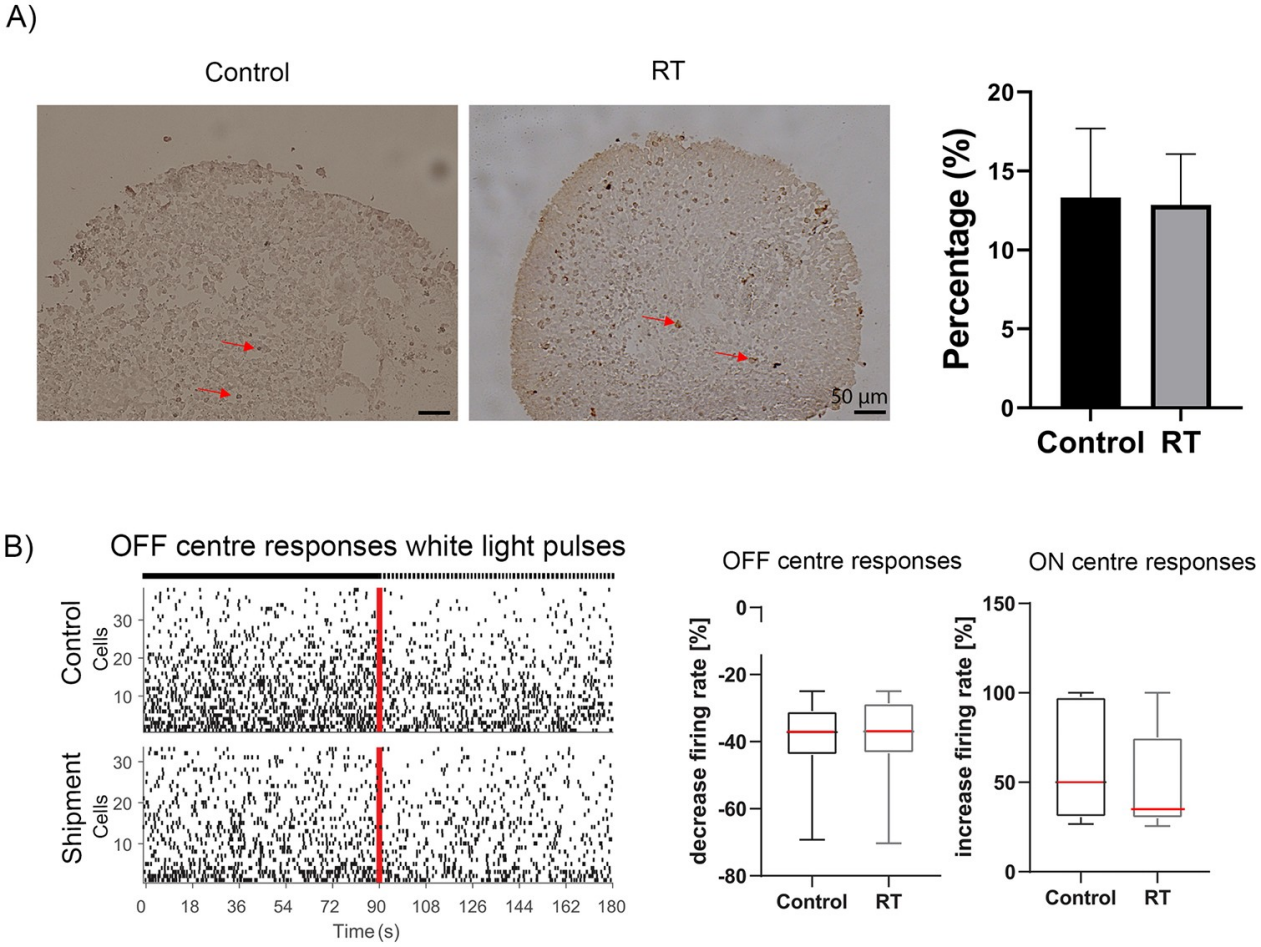
Apoptotic cells and functionality of day 135 recovered shipped retinal organoids. (A) Brightfield images of apoptotic and necrotic cells were detected in control (left image) and shipped organoids (right image), stained with dark brown colour, indicated by red arrows. No significant differences were observed between control and shipped organoids (p = 0.9355). (B) Spike raster plots (top) and firing rate histogram (bottom) from putative OFF retinal ganglion cells (RGCs) of control and shipped d135 organoids revealed that their spiking activity decreased after white light pulses (WLP). Each row in the raster plot (y-axis) represents a different RGC and each vertical bar represents a spike from the corresponding RGC. The red line illustrates the stimulus onset whereas the left half before indicates the spontaneous activity before WLP exposure and the right half when exposed to WLP. Box plot indicates the decreased firing rate (in %) of putative OFF RGCs (top) and ON RGC (bottom) in control and RT condition. In both cases, no significant differences were observed (Mann Whitney test; p = 0.77 for OFF RGCs and p = 0.18 for ON RGCs). The box plot shows the median (red line).

Bright field images of day 135 organoids were taken to assess the morphology and neuroepithelial thickness. The structure and neuroepithelial thickness of shipped organoids revealed no significant differences compared to day 135 control organoids (Fig 3B and 3C). Immunohistochemical analysis revealed no significant differences in percentage and position of Recoverin positive photoreceptors (Fig 3D and 3E, S4 Fig). A small percentage of cells identified in the middle layer of day 135 organoids was positive for AP2α cells in both conditions, indicating the presence of amacrine cells (Fig 3D and 3E, S4 Fig). Additionally, cells positive for HuC/D, a marker of amacrine and ganglion cells, were present in the centre of the retinal organoids in both conditions (Fig 3D, S4 Fig). The presence of ganglion cells was also detected by SNCG marker (Fig 3D, S4 Fig). ARL13B was detected at the apical side above photoreceptor cell nuclei of control and shipped organoids, indicating the formation of connecting cilia (Fig 3D, S4 Fig). Vimentin and Sox9 and Prox1 immunopositive cells were present in both conditions, indicating the presence of Müller glial and horizontal cells respectively (Fig 3D, S4 Fig). The expression of Rod photoreceptors was detected by Rhodopsin in both conditions (Fig 3D, S4 Fig). Overall, the quantification analysis of all retinal markers showed no significant differences between shipped and control organoids (Fig 3D, S4 Fig). Additionally, the number of dead cells in control and RT condition was detected by Tunel assay, showing no significant differences between the two groups (Fig 4A).

To validate if the physiological functionality of retinal organoids was affected by the shipment, multielectrode recordings were performed for day 135 organoids that were shipped and further placed in incubator for another 45 days. In both conditions putative OFF retinal ganglion cells (RGCs) revealed a decrease in their spiking activity after white light pulses (WLP) (Fig 4B), indicating no significant difference between the two groups (Fig 4B; p = 0.77). In the same way, presumed ON RGCs which increase their spiking activity when exposed to WLP showed no differences between both conditions (Fig 4B; p = 0.18). Taken together, these results support that day 135 retinal organoids can retain their structure, morphology and function following shipment at RT conditions.

#### 3.2.2. Comparison of control and shipped organoids at day 160

**Fig 5:**
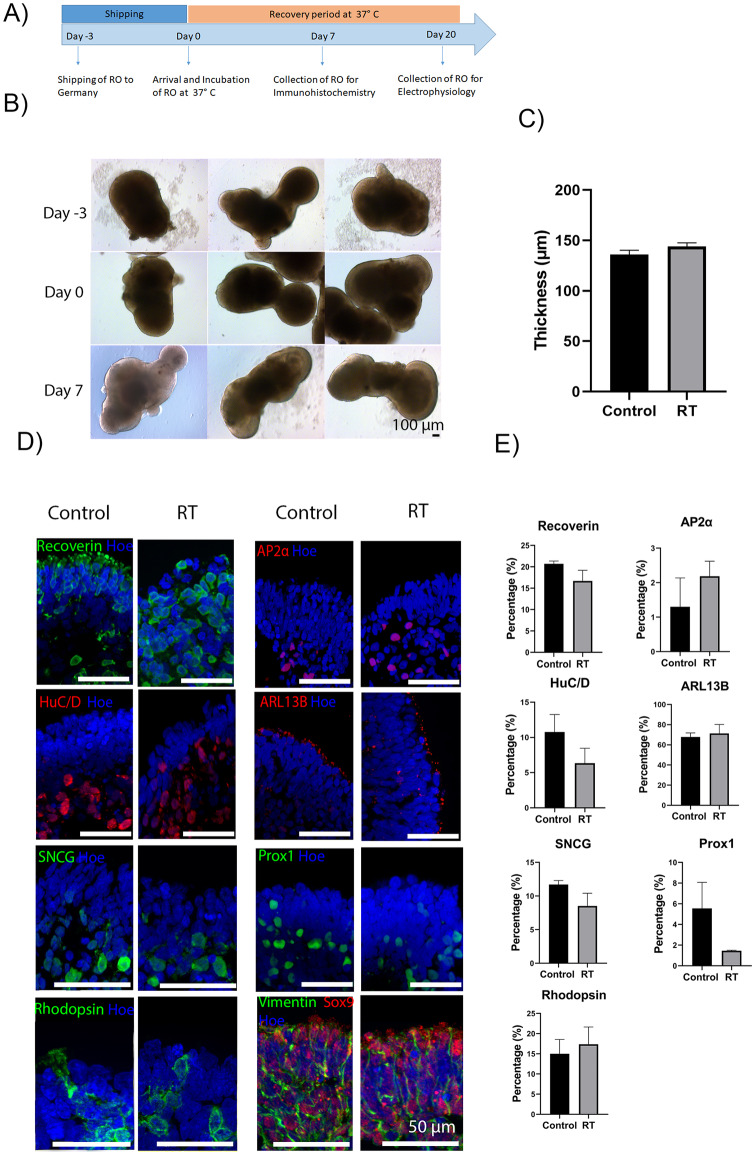
Characterisation of day 160 retinal organoids after shipment. (A) Schematic diagram of shipment experiment. (B) Bright field images of day 160 hiPSCs-derived retinal organoids were captured on day -3, representing the organoids before shipping, day 0 arrival of shipped organoids, and day 7 represents the recovery period of the organoids in a humidified environment at 37°C with 5% CO_2_. Scale bars = 100 μm. (C) Measurement of neuroepithelial thickness of day 160 shipped and control organoids. The data are shown as mean ± SEM and the thickness is measured in μm. An unpaired t-test was performed to estimate differences in the thickness of the apical layer between the control and RT organoids, indicating no significant differences between the two groups, p = 0.1328. (D) Expression of retinal marker for photoreceptors (Recoverin, green), amacrine cells (AP2α, red), amacrine and ganglion cells (HuC/D, red), ganglion cells (SNCG, green), connecting cilium (ARL13B red), Müller cells (Vimentin–green, Sox9—red), Rod photoreceptors (Rhodopsin, green) and horizontal cells (Prox1, green) in control and RT condition. Nuclei were counterstained with Hoechst (Hoe, blue). Scale bar = 50 μm. (E) Quantification of retinal marker protein expression for Recoverin (p = 0.2648) AP2α (p = 0.4005), HuC/D (p = 0.1785), SNCG (p = 0.1594), ARL13B (p = 0.6913), Rhodopsin (p = 0.7023) and Prox1 (p = 0.1785) revealed no significant difference in the percentage of immunoreactive-positive cells between control and shipped organoids. Data are shown as mean ± SEM. 3–5 representative images per cell marker were quantified per condition.

**Fig 6:**
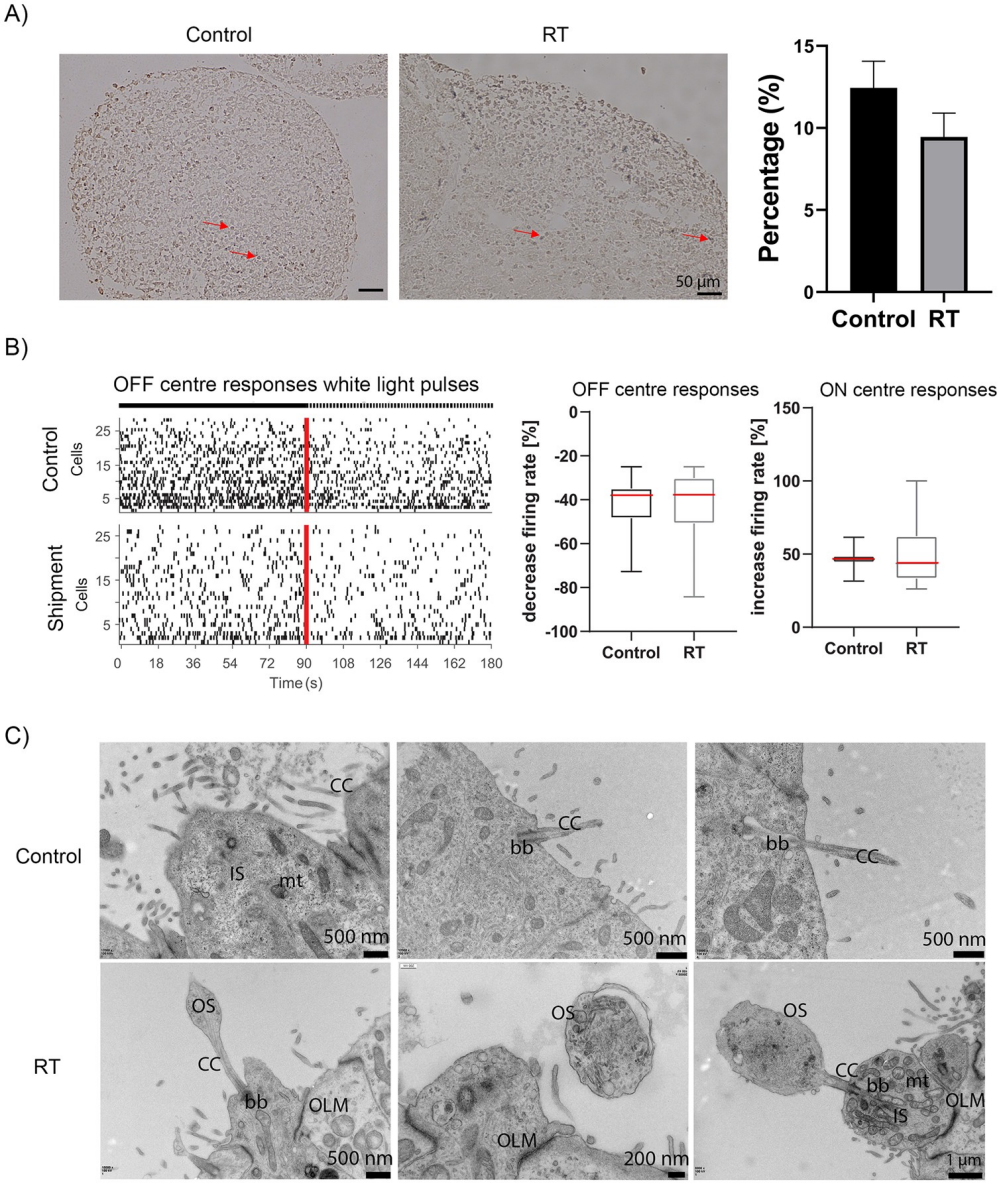
Apoptotic cells, functionality and ultrastructural characteristics of day 160 recovered shipped retinal organoids. (A) Brightfield images illustrate with red arrows the apoptotic and necrotic cells in control (left image) and shipped organoids (right image), stained with dark brown colour. No significant differences were observed between control and shipped day 165 organoids (p = 0.1888). (B) Spike raster plots (top) and firing rate histogram (bottom) from putative OFF RGCs of control and shipment group (day 160) showed a decreased spiking activity after white light pulses (WLP). Box plot revealed the decreased firing rate (in %) of putative OFF RGCs (top) and ON RGC (bottom) in control and RT condition. There are no significant differences between both conditions (Mann Whitney test; p = 0.56 for OFF RGCs and p = 0.86 for ON RGCs). The box plot shows the median (red line). (C) Ultrastructural analysis of day 160 retinal organoids revealed the presence of connecting cilium (CC), basal body (bb), photoreceptor outer segments (OS), photoreceptors possessing inner segments (IS), outer limiting membrane (OLM) and mitochondria (mt) in both shipped and control organoids.

Next, we used retinal organoids at day 160 of differentiation to test whether organoids at a more advanced stage of differentiation would be affected by the shipment (Fig 5A). The morphology and the neuroepithelial thickness of shipped and control organoids showed no significant differences (Fig 5B and 5C). Immunohistochemistry analysis revealed the presence of Recoverin positive photoreceptors in the apical layer in both control and shipped groups (Fig 5D, S5 Fig). AP2α positive cells were found in the middle layer, indicating the presence of amacrine cells (Fig 5D, S5 Fig). HuC/D and SNCG was expressed in the basal layer of both control and shipped retinal organoids (Fig 5D, S5 Fig). A small percentage of Prox1 positive cells (horizontal cells) was observed in both shipped and control organoids (Fig 5D and 5E, S5 Fig). Müller glia cells identified by Vimentin and Sox9, were also found to span the length of the control and shipped organoids (Fig 5D, S5 Fig). Additionally, ARL13B positive cilia were observed in the apical layer above photoreceptor nuclei in both groups, suggesting the presence of connecting cilia and the beginning of outer segments formation (Fig 5D, S5 Fig). Rod photoreceptors were detected by Rhodopsin in the apical layer, in both conditions (Fig 5D, S5 Fig). Quantification of all markers revealed no significant differences in the percentage of positive cells between the two conditions (Fig 5E). Tunel assay was used to detect the number of apoptotic and necrotic cells in control and shipped organoids, which are indicated by red arrows, showing no significant differences between the two conditions (Fig 6A). Multielectrode recordings were carried out to assess the impact of organoid shipment on their physiological function in response to light stimulation. Following 20 days of incubation after shipping, the shipped and control retinal organoids exhibited similar retinal ganglion cells responses. For example, presumed OFF centre RGC responses showed a reduction in their spiking activity in both conditions, as well as putative ON centre RGC responses which increase their spiking activity after light exposure (Fig 6B). There was no significance difference between the control and the shipped group (OFF centre responses: p = 0.56, ON centre responses p = 0.86; Fig 6B), which indicates that shipment of day 160 retinal organoids has no effect on their function. Together these data suggest that short term storage and shipment of retinal organoids does not affect their structure, morphology or function.

TEM of control and shipped organoids at day 160 revealed the presence of organized photoreceptor-like ultrastructural features including the photoreceptor OS, IS, connecting cilium and outer limiting membrane residing in the apical layer of retinal organoids (Fig 6C). In addition, basal body located at the base of connecting cilium and photoreceptor IS-rich in mitochondria were observed (Fig 6C). These results confirmed the previous findings and indicated no ultrastructural differences between RT and control organoids.

## 4. Discussion

In the last decade, improvements in the generation of retinal organoids from hiPSCs have been reported in several key studies [1, 2, 15], leading to generation of 3D aggregates which resemble the adult retina in terms of cell type composition and layering and to some extent the electrophysiological function [1, 4, 16]. Generation of organoids requires skills and expertise, which may not be available in every lab; thus validation of most optimal transport conditions which maintain their structure and function is very important for ensuring worldwide applications in drug discovery and cell therapy based studies.

In this study we investigated the impact of five day storage and three day shipment at RT on retinal organoids. Our results indicated that retinal organoids generated from two different hiPSC lines retained their morphology at RT conditions following subsequent recovery period. Moreover, the study confirmed that shipment of retinal organoids at different developmental stages had no effect on the structure, morphology, biological activity or physiological function, thus providing an optimal solution for increasing their applications to a large number of labs.

Using special containers that control and maintain the environment at ambient temperature prevents temperature fluctuations and thus minimises the risk of cell death or functional impairment of tissues. Importantly, maintaining the organoids at RT conditions facilitates a quick recovery period, enabling experiments and tests to be performed quicker, avoiding lost time, structural damage and toxicity effects associated with cryopreservation [17].

In conclusion our data indicate that RT shipment of retinal organoids using a controlled environment container maintains their structural features and biological activity. Therefore, live shipment at RT could provide the method of choice to enable collaboration between laboratories. The organoid recovery time is short and the costs of transportation low, making this an optimal and cost effective solution for broad application in research and cell based therapies.

## Supporting information

S1 Fig Shipment of retinal organoids. (TIF)

S2 Fig Schematic diagram of the differentiation process. (TIF)

S3 Fig Immunohistochemical analysis of retinal markers of control and RT retinal organoids shown in Fig 1 in split channels, after 15 days of recovery from storage at RT for 5 days. Expression of photoreceptors (Recoverin, green) and amacrine and ganglion cells (HuC/D, red), Müller cells (Vimentin–green, Sox9 -red), bipolar cells (PKCα, red), Rod photoreceptors (Rhodopsin, green), ganglion cells (SNCG, green), amacrine cells (AP2α, red), and S cone photoreceptors (Opsin SW, green), L/M cone photoreceptors (Opsin MW/LW, green) and horizontal cells (Prox1, green). Nuclei are counterstained with Hoechst (Hoe, blue). Scale bar = 50 μm.
(TIF)

S4 Fig Immunohistochemical analysis of retinal markers of control and shipped day 135 retinal organoids shown in Fig 3 in split channels. Expression of retinal markers in both control and shipped retinal organoids revealed the presence of photoreceptors (Recoverin, green), Rod photoreceptors (Rhodopsin, green), amacrine cells (AP2α, red), amacrine and ganglion cells (HuC/D, red), ganglion cells (SNCG), connecting cilium (ARL13B red), Müller cells (Vimentin–green, Sox9 –red) and horizontal cells (Prox1, green). Nuclei were counterstained with Hoechst (Hoe, blue). Scale bar = 50 μm.
(TIF)

S5 Fig Immunohistochemical analysis of retinal markers of control and shipped day 160 retinal organoids shown in Fig 5 in split channels. Expression of retinal marker for photoreceptors (Recoverin, green), amacrine cells (AP2α, red), amacrine and ganglion cells (HuC/D, red), ganglion cells (SNCG, green), connecting cilium (ARL13B red), Müller cells (Vimentin–green, Sox9—red), Rod photoreceptors (Rhodopsin, green) and horizontal cells (Prox1, green) in control and RT condition. Nuclei were counterstained with Hoechst (Hoe, blue). Scale bar = 50 μm.
(TIF)

S1 Table List of antibodies used for immunohistological analysis. (DOCX)

S2 Table List of secondary antibodies used for immunohistological analysis. (DOCX)
