# Supplementary figures and images for: Room temperature shipment does not affect the biological activity of pluripotent stem cell-derived retinal organoids
Source: PLoS One. 2020 Jun 1;15(6):e0233860. doi: 10.1371/journal.pone.0233860 (PMC7263587; doi:10.1371/journal.pone.0233860)

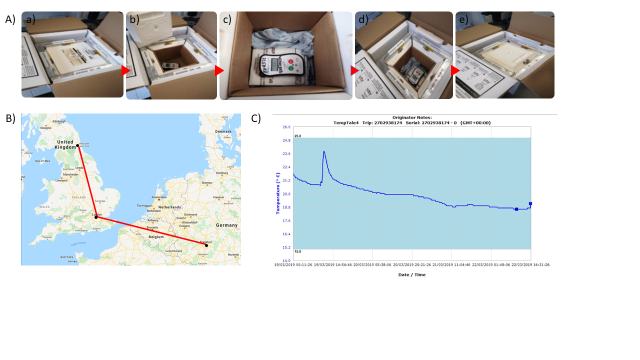

Supplement: S1 Fig — (TIF) [file pone.0233860.s001.tif]

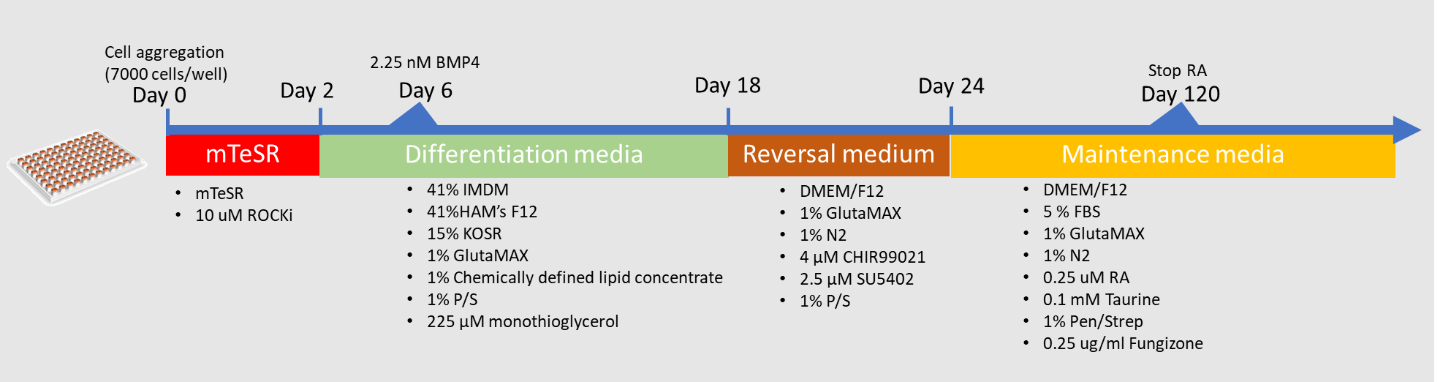

Supplement: S2 Fig — (TIF) [file pone.0233860.s002.tif]

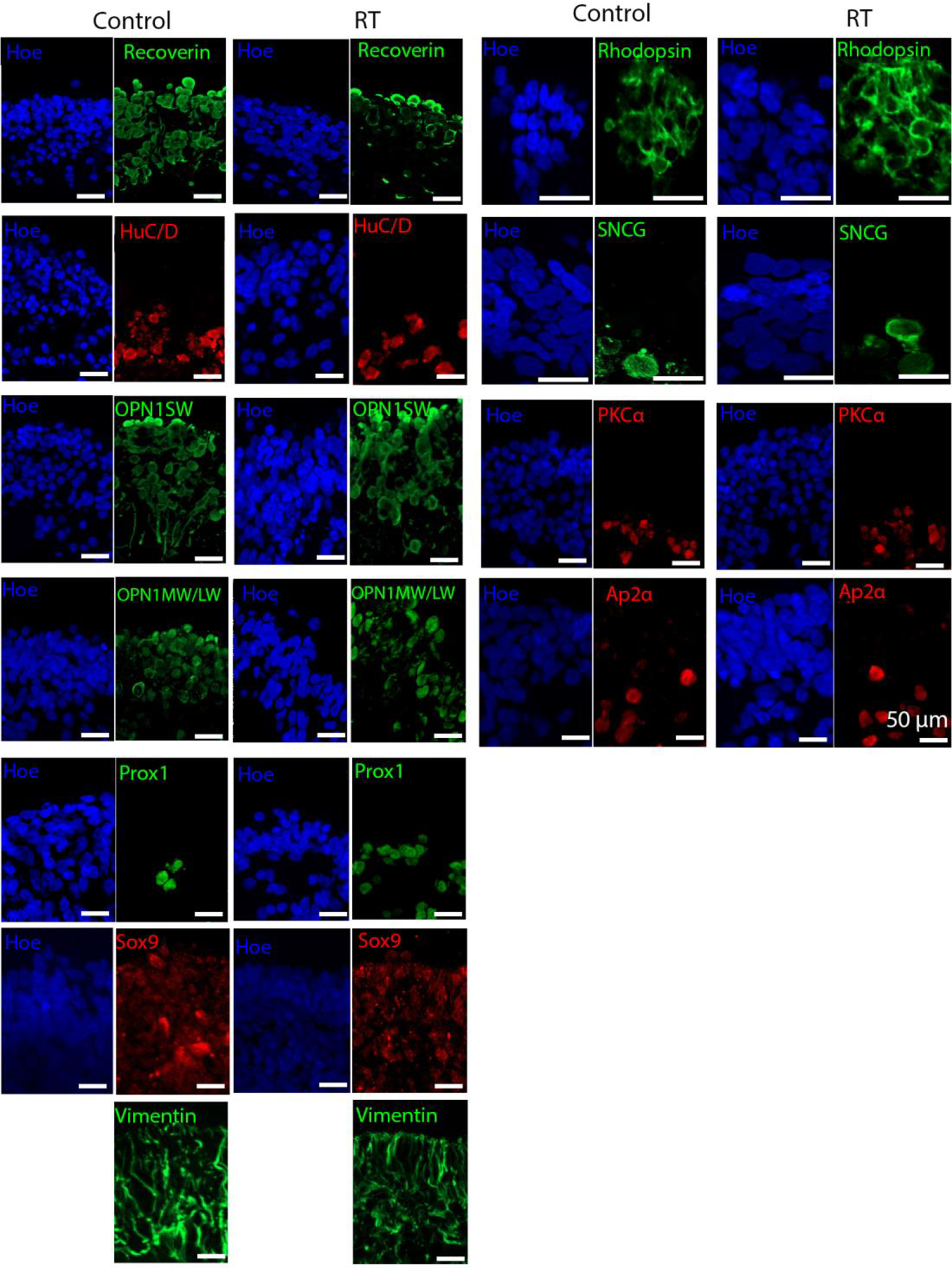

Supplement: S3 Fig — Expression of photoreceptors (Recoverin, green) and amacrine and ganglion cells (HuC/D, red), Müller cells (Vimentin–green, Sox9 -red), bipolar cells (PKCα, red), Rod photoreceptors (Rhodopsin, green), ganglion cells (SNCG, green), amacrine cells (AP2α, red), and S cone photoreceptors (Opsin SW, green), L/M cone photoreceptors (Opsin MW/LW, green) and horizontal cells (Prox1, green). Nuclei are counterstained with Hoechst (Hoe, blue). Scale bar = 50 μm. (TIF) [file pone.0233860.s003.tif]

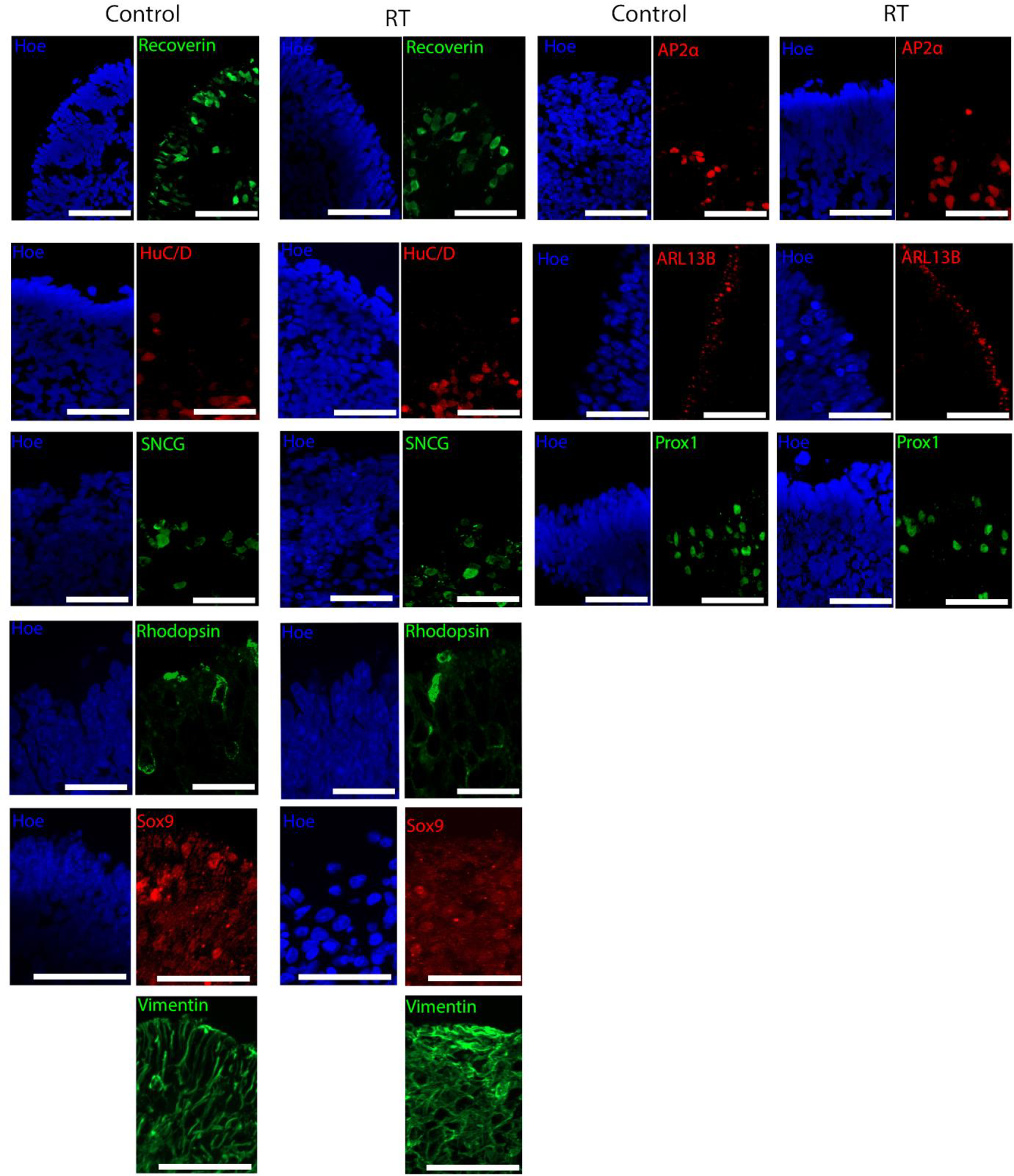

Supplement: S4 Fig — Expression of retinal markers in both control and shipped retinal organoids revealed the presence of photoreceptors (Recoverin, green), Rod photoreceptors (Rhodopsin, green), amacrine cells (AP2α, red), amacrine and ganglion cells (HuC/D, red), ganglion cells (SNCG), connecting cilium (ARL13B red), Müller cells (Vimentin–green, Sox9 –red) and horizontal cells (Prox1, green). Nuclei were counterstained with Hoechst (Hoe, blue). Scale bar = 50 μm. (TIF) [file pone.0233860.s004.tif]

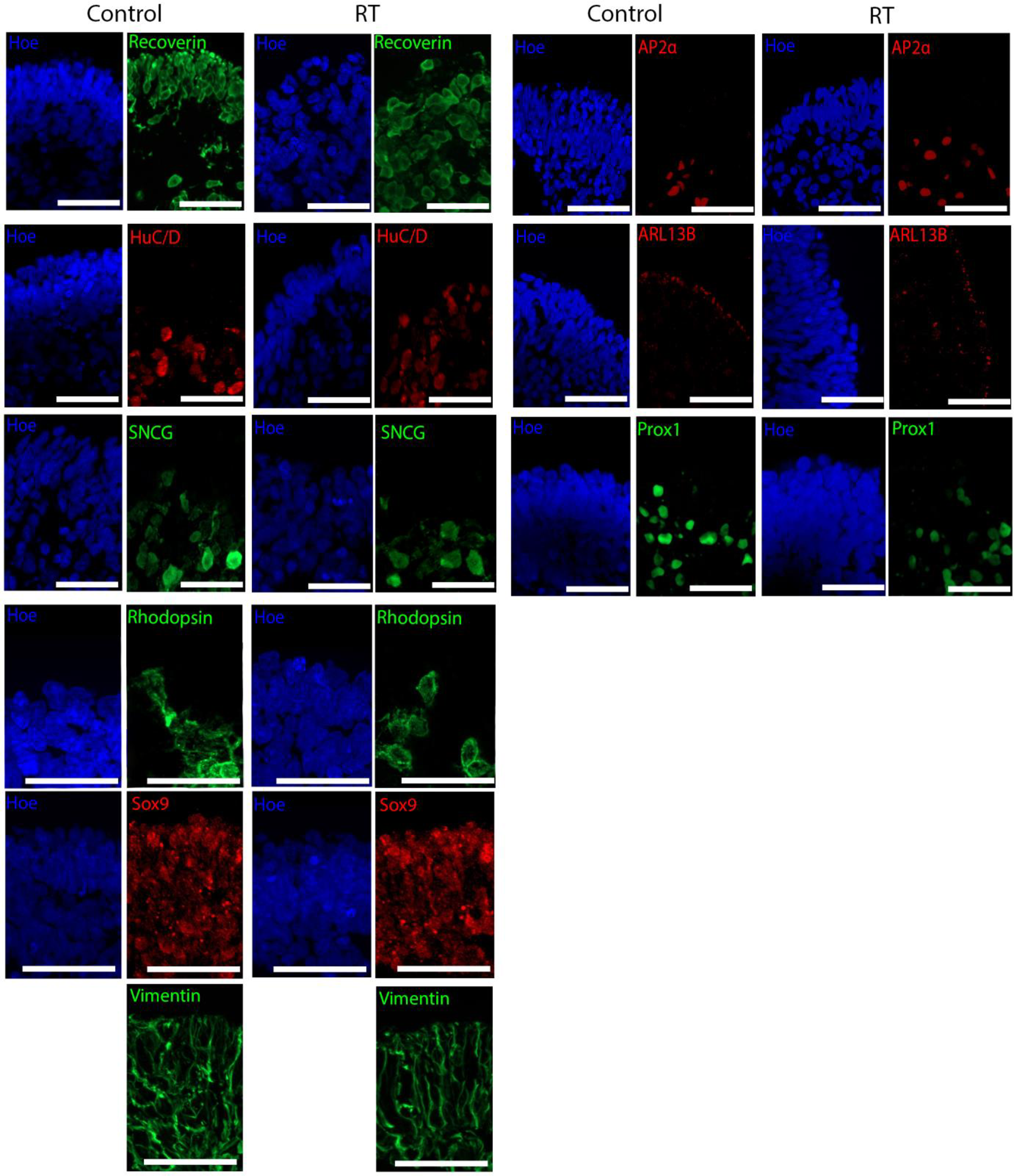

Supplement: S5 Fig — Expression of retinal marker for photoreceptors (Recoverin, green), amacrine cells (AP2α, red), amacrine and ganglion cells (HuC/D, red), ganglion cells (SNCG, green), connecting cilium (ARL13B red), Müller cells (Vimentin–green, Sox9—red), Rod photoreceptors (Rhodopsin, green) and horizontal cells (Prox1, green) in control and RT condition. Nuclei were counterstained with Hoechst (Hoe, blue). Scale bar = 50 μm. (TIF) [file pone.0233860.s005.tif]
