# Supplementary material for: Room temperature shipment does not affect the biological activity of pluripotent stem cell-derived retinal organoids
Source: PLoS One. 2020 Jun 1;15(6):e0233860. doi: 10.1371/journal.pone.0233860 (PMC7263587; doi:10.1371/journal.pone.0233860)
Supplement: S1 Table — (DOCX) [file pone.0233860.s006.docx]

**S1 Table. List of antibodies used for immunohistological analysis.**

| Antibody | Conjugate/Tissue | Host | Source | Cat. No. | Dilution |
| --- | --- | --- | --- | --- | --- |
| Recoverin | photoreceptors and midget OFF bipolar cells | Rabbit | Millipore | AB5585 | 1:1000 |
| HuC/D | Amacrine and retinal ganglion cells | Mouse | Invitrogen | A21271 | 1:200 |
| CRALBP | RPE and Müller cells | Mouse | Abcam | ab15051 | 1:100 |
| Prox 1 | Horizontal cells | Rabbit | Millipore | AB5475 | 1:1000 |
| Opsin MW/LW | L/M cone photoreceptors | Rabbit | Millipore | AB5405 | 1:200 |
| ARL13B | Connecting cilium | Rabbit | Abcam | ab83879 | 1:250 |
| Opsin SW | S cone photoreceptors | Rabbit | Abcam | AB5407 | 1:200 |
| Ap2α | Amacrine cells | Mouse | Santa Cruz | sc-12726 | 1:200 |
| *PKCa* | Bipolar Cells | Mouse | BD Transduction Laboratories, | 610107 | 1:200 |
