# Supplementary material for: Room temperature shipment does not affect the biological activity of pluripotent stem cell-derived retinal organoids
Source: PLoS One. 2020 Jun 1;15(6):e0233860. doi: 10.1371/journal.pone.0233860 (PMC7263587; doi:10.1371/journal.pone.0233860)
Supplement: S2 Table — (DOCX) [file pone.0233860.s007.docx]

**S2 Table. List of secondary antibodies used for immunohistological analysis.**

| Antibody | Host | Source | Cat. No. | Dilution |
| --- | --- | --- | --- | --- |
| Gt a-r Alexa (488) | Jackson ImmunoResearch | Goat | 111545144 | 1:800 |
| Gt a-m Cy3 | Jackson ImmunoResearch | Goat | 115165003 | 1:800 |
